# Ophthalmic manifestations and management considerations for emerging chemical threats

**DOI:** 10.3389/ftox.2023.1281041

**Published:** 2023-10-18

**Authors:** Bryant A. Menke, Christine Ryu, Grant A. Justin, Rao V. Chundury, Brent R. Hayek, Matthew R. Debiec, Steven Yeh

**Affiliations:** ^1^ Department of Ophthalmology, Truhlsen Eye Institute, University of Nebraska Medical Center, Omaha, NE, United States; ^2^ Walter Reed National Military Medical Center, Washington, DC, United States; ^3^ National Strategic Research Institute, University of Nebraska Medical Center, Omaha, NE, United States; ^4^ North Georgia Eye Clinic, Gainesville, GA, United States; ^5^ Global Center for Health Security, University of Nebraska Medical Center, Omaha, NE, United States

**Keywords:** chemical threats, sulfur mustard, lewisite, sarin, VX, cyanide, hydrofluoric acid

## Abstract

Chemical agents have been utilized for centuries in warfare and pose a health threat to civilians and military personnel during armed conflict. Despite treaties and regulations against their use, chemical agent exposure remains a threat and measures to understand their effects and countermeasures for systemic and organ-specific health are needed. Many of these agents have ocular complications, both acute and chronic. This mini-review focuses on key chemical agents including vesicants (mustards, lewisite), nerve agents (sarin, VX), knockdown gasses (hydrogen cyanide), and caustics (hydrofluoric acid). Their ophthalmic manifestations and appropriate treatment are emphasized. Acute interventions include removal of the source and meticulous decontamination, as well as normalization of pH to 7.2–7.4 if alteration of the ocular pH is observed. Besides vigorous lavage, acute therapies may include topical corticosteroids and non-steroid anti-inflammatory therapies. Appropriate personal protective equipment (PPE) and strict donning and doffing protocols to avoid healthcare provider exposure are also paramount in the acute setting. For more severe disease, corneal transplantation, amniotic membrane graft, and limbal stem cell transplantation may be needed. Orbital surgery may be required in patients in whom cicatricial changes of the ocular surface have developed, leading to eyelid malposition. Multidisciplinary care teams are often required to handle the full spectrum of findings and consequences associated with emerging chemical threats.

## 1 Introduction

Chemical agents have been used in warfare as early as 600 BCE when the Athenian military tainted the water supply of a sieged city. In the modern era, the first large-scale chemical weapons were used during World War I at the Second Battle of Ypres where chlorine gas resulted in 6,000–7,000 casualties (Fitzgerald, 2008; Mayor, 2003). As chemicals can easily immobilize troops with relatively low costs and effort compared to arms-based tactics, there is a risk for use by adversaries who do not adhere to the multiple treaties limiting their use, such as the Geneva Protocol and Chemical Weapons Convention. Recently, chemical warfare was used in 2018 by the Syrian Air Force causing nearly 70 deaths (Omar, 2020).

The Centers for Disease Control and Prevention categorize chemical agents into the following groups: vesicants (blister agents), nerve agents, choking/lung agents, caustics, blood agents, incapacitating agents, metals, riot control agents, toxic alcohols, and biotoxins (CDC, 2022). These agents may be titrated depending on the level of attempted damage. In addition to their ability to temporarily disarm opposing forces or cause fatality at high concentrations, many of these agents have significant long-term effects. The potential for chronic effects underscores the importance of understanding their consequences, including systemic and organ-specific findings, as well as the appropriate management paradigms for front-line healthcare providers.

This focused review covers vesicants (mustards, lewisite), nerve agents (sarin, VX), knockdown gasses (hydrogen cyanide), and caustics (hydrofluoric acid). Their ophthalmic manifestations and appropriate treatment are emphasized. A brief summary of each agent discussed can be found in Table 1.

**TABLE 1:** Summary of chemical agents, systemic and ocular findings and management.

Agent	Odor and color	Systemic effects	Ocular effects	Management^a^
Vesicants				
Sulfur mustard	Odorless, garlic, onion, mustard	Pulmonary: Acute rhinitis, pharyngitis, tracheitis, bronchitis; dyspnea, pulmonary edema, alveolar hemorrhage, chronic bronchitis, bronchial asthma, recurrent respiratory infections	Acute: Conjunctival injection, keratitis, corneal edema, uveitis, blepharospasm, corneal neovascularization, corneal perforation	Sodium thiosulfate
		Skin: Erythema, bullae, ulceration, scar formation		Pulmonary: humidified oxygen, N-acetylcysteine, rehydration, intensive respiratory support
	Colorless, may be yellow-brown	Lethal dose in 50% of the population (LD50) estimates: 100 mg/kg. dermal; 0.7 mg/kg oral	Chronic: persistent conjunctival irritation and photophobia, corneal opacities, ulceration, band keratopathy, corneal melting with neovascularization	Ocular: mydriatics, sunglasses, petroleum jelly
				Consideration for limbal stem cell therapy or corneal keratoplasty^b^
Lewisite	Odor of geraniums	Respiratory: rapid burning, pain, irritation, pneumonitis, respiratory failure, severe pulmonary edema, development of malignant lesions	Acute: Immediate pain and irritation, vesication of corneal epithelium, full-thickness keratocytosis, corneal neovascularization, uveitis, miosis	Chelating agent: British Anti-Lewisite (2,3-dimercapto-1-propanol)
		Skin: instantaneous erythema and burning, edema and bullae formation, necrosis, development of malignant lesions		
	If pure, colorless. If impure, amber to black color	“Lewisite Shock”—increased vascular permeability, hypotension, multi-organ failure	Chronic: corneal opacification, corneal perforation, blindness	
		LD50 estimate: 40 mg/kg dermal		
Nerve agents				
Sarin	Odorless, colorless	Systemic: Diarrhea, micturition, bronchospasm, bradypnea, increased respiratory secretions, bradycardia, dysrhythmia	Acute: Lacrimation, miosis, ciliary muscle spasm, blurred vision, myopia, ocular pain, headaches, blunted light reflex	Atropine followed by pralidoxime infusion
		Neurologic: confusion, altered mental status, central apnea, seizure. Long term can have insomnia, depression, anxiety, impaired memory		
VX	Odorless, amber when liquid	Sarin LD50 estimate: 100–500 mg dermal	Chronic: No known long-term sequelae	
		VX LD50 estimates:10 mg dermal; 25–30 mg inhalation		
Knockdown gases				
Hydrogen	Bitter almond odor	Systemic: tachycardia and tachypnea early, arrhythmias, bradycardia, hypotension, apnea late manifestations	Acute: mydriasis, decreased visual acuity due to optic neuritis	Rescue breaths contraindicated
Cyanide	Colorless gas, colorless or pale blue liquid	Neurologic: headache, confusion, dizziness, seizures, coma	Chronic: optic disc atrophy, retrobulbar visual tract lesions	Hydroxocobalamin preferred over sodium nitrate and thiosulfate combination therapy
		LD50 estimates: 100 mg/kg dermal; 1.52 mg/kg oral; 1.0 mg/kg oral		
Caustics				
Hydrofluoric	Pungent irritating odor	Skin: Pain (classically out of proportion to exam), ulceration and necrosis, potential involvement of underlying bones and tendons	Acute: immediate or delayed pain, conjunctivitis, edema, erosion, sloughing, ulceration of corneal epithelium	Consider 1% calcium ocular irrigation^b^, calcium gluconate gel for dermal burns
	Colorless	Respiratory: pain, inflammation of respiratory mucosa, ulceration, nasal septal perforation, laryngitis, tracheitis, bronchitis. Pulmonary edema and hemorrhage, pneumothorax		
Acid		Gastrointestinal: nausea, vomiting, severe pain, melena, hematemesis due to ulceration, perforation	Chronic: Corneal opacifications, visual acuity impairment, photophobia, globe perforation, glaucoma, uveitis, keratitis sicca	Hexafluorine shown to be effective in small case series^b^
		Cardiovascular: fatal arrhythmias		
		LD50 estimate: 20 mg/kg oral		

^a^ The most important immediate consideration for every agent is decontamination with clothing removal, cleaning the contaminated skin, ocular rinsing, etc.

^b^ Variable results in the literature for treatment.

## 2 Sulfur mustard

Sulfur mustard, also called mustard gas or its military designation, HD, is a vesicant. Approximately 77% of the gas injuries during World War I were due to sulfur mustard (Ganesan, 2010). More recently, the Iran-Iraq war saw its widespread use (Smith and Dunn, 1991). Although chemical damage begins minutes after contact, manifestations of toxicity appear after a latency period, lasting up to 12 h with exposures under 60 mg min/m^3^ or under 3 h with exposures over 60 mg min/m (Institute of Medicine Committee on the Survey of the Health Effects of Mustard Gas and Lewisite, 1993; Gates and Moore, 1946; Mandel and Gibson, 1917; Balali-Mood and Hefazi, 2005). The latency period is also dependent on the ambient temperature, with hot, humid environments decreasing the latency period significantly. Within the respiratory system, acute exposure leads to acute rhinopharyngeotracheobronchitis and vacuolization of respiratory epithelium, resulting in dyspnea and alveolar hemorrhage (Devereaux et al., 2002; Khateri et al., 2003). Skin manifestations range from pain and erythema to deep bullae, which can ulcerate (Poursaleh et al., 2012). The most common chronic pulmonary complication is chronic bronchitis, seen nearly half of exposures, as well as recurrent respiratory infections and bronchial asthma (Emad and Rezaian, 1997).

### 2.1 Ocular complications

The acute ocular effects range in severity from conjunctival injection to corneal edema, corneal opacities, keratitis, uveitis, and blepharospasm (Balali-Mood and Hefazi, 2005). Given the corneal epithelium’s high metabolic rate, the eyes are ten times more sensitive to sulfur mustard injury than other primary target organs (Institute of Medicine Committee on the Survey of the Health Effects of Mustard Gas and Lewisite, 1993). In addition, sulfur mustard is lipophilic, which increase absorption through the tear film (Solberg et al., 1997). Acute ocular symptoms typically occur after exposures of at least 50 mg min/m^3^ (Goverman et al., 2014). The chronic effects of exposure appear to be related to the extent of initial exposure, route of contact, whether removed from exposure and treated, and individual factors, such as age, sex, and health status (Amini et al., 2020). Up to 83% of patients report chronic ocular symptoms, most commonly persistent conjunctival irritation and photophobia (Khateri et al., 2003; Namazi et al., 2009). However, more severe chronic symptoms include moderate corneal opacities and ulceration, corneal edema, band keratopathy, and corneal melting with neovascularization are seen in 10% of exposures (Namazi et al., 2009). The constellation of chronic corneal findings is termed *mustard gas keratopathy*, in which the extent of corneal damage may lead to months of hospitalization or blindness (Daryabari et al., 2022). About 0.5% of patients with severe sulfur mustard injuries later develop a delayed, recurrent keratopathy which can happen 8–40 years after initial injury (Institute of Medicine Committee on the Survey of the Health Effects of Mustard Gas and Lewisite, 1993; Solberg et al., 1997).

### 2.2 Therapeutic considerations

Immediate decontamination is the most important initial management. Please refer to the “Therapeutic Approach to Chemical Exposures” section for decontamination and ocular rinsing procedures. Skin absorption occurs in 2 min, so effective decontamination within those 2 min can prevent the effects of sulfur mustard. Additionally, sodium thiosulfate can be used for systemic effects (Etemad et al., 2019). Humified oxygen, N-acetylcysteine, rehydration, and more invasive respiratory support as needed are the mainstay of treatment for pulmonary symptoms.

For ocular symptoms, mydriatics can be used for pain and ciliary muscle spasms, dark sunglasses for photophobia, and petroleum jelly or antibiotic ophthalmic ointment for the prevention of lid adhesions (Panahi et al., 2017). Skin lesions should be kept clean to prevent secondary infections. For cicatricial eyelid changes, corrective surgeries such as ectropion repair can be done.

## 3 Lewisite

Lewisite is an arsenic-based chemical that was once a primary agent but is now used as an adjunct to increase the environmental persistence of sulfur mustard (McNutt and Hamilton, 2015). Unlike sulfur mustard, lewisite symptoms appear within minutes of exposure, making it a less effective agent (Gates et al., 1946). The acute symptoms are similar to that of sulfur mustard, with instantaneous erythema and burning of the skin with later edema and bullae formation that is maximal at 36–48 h (Institute of Medicine Committee on the Survey of the Health Effects of Mustard Gas and Lewisite, 1993). Ulceration and necrosis can occur in skin with higher levels of exposure. There are reports of malignant lesions appearing in the areas of previous exposure in both the skin and respiratory tract (Doi et al., 2011). In the respiratory tract, acute exposure leads to rapid burning, pain, and irritation, with more severe exposures leading to pneumonitis, respiratory failure, and severe pulmonary edema (Manzoor et al., 2020). Lewisite can be lethal with acute toxicity due to dermal absorption and systemic distribution, referred to as *lewisite shock*, which manifests as a result of increased vascular permeability and subsequent third-spacing with damage to the biliary tree, liver, gallbladder, and lungs (Chauhan et al., 2008). Multiorgan failure including renal and liver failure can lead to death (Srivastava et al., 2018).

### 3.1 Ocular complications

The acute ocular effects of lewisite include immediate eye pain, irritation, lacrimation, blepharospasm, and chemosis that peaks 4–6 h after exposure (Institute of Medicine Committee on the Survey of the Health Effects of Mustard Gas and Lewisite, 1993). With high doses of vapor exposure, vesication of corneal epithelium, full-thickness keratocytosis, and neovascularization can occur. Lewisite has also been shown to cause severe uveitis and miosis due to penetration into the eye (Institute of Medicine Committee on the Survey of the Health Effects of Mustard Gas and Lewisite, 1993). The chronic effects of ocular exposure include corneal opacification, corneal perforation, and blindness (Tewari-Singh et al., 2016). Severe eyelid blistering and ulceration can lead to scarring and lid malposition.

### 3.2 Therapeutic considerations

The most crucial acute treatment is removal from the contaminated area in addition to decontamination measures (CDC, 2023). Unlike sulfur mustard, lewisite has a specific chelating antidote for lewisite, 2,3-dimercapto-1-propanol (British Anti-Lewisite), that has been shown to reduce systemic injury from lewisite exposure (Vilensky and Redman, 2003). However, ophthalmic formulations are not currently available. Eyelid wounds from lewisite are treated similarly to those from sulfur mustard: hygiene, monitoring for and prevention of secondary infections, and surgical treatment of ocular surface scarring and eyelid malposition.

## 4 Nerve agents–sarin and VX

Nerve agents are irreversible acetylcholinesterase inhibitors, similar to pesticides, leading to cholinergic hyperactivity (Mukherjee and Gupta, 2020). These agents are sub-classified into two main classes, the G-agents and the V-agents. The G-agents, such as sarin, are more volatile, making them less stable and less effective than the V-agents, such as VX (Radilov et al., 2009). Given its low volatility, VX has a long environmental persistence, making it a more lethal agent. Recently, sarin attacks were noted in Syria in 2013, and a VX attack occurred in Malaysia in 2017. The latter of which resulted in the death of Kim Jong-Nam, the brother of Kim Jong-Un (Chai et al., 2017; Rosman et al., 2014). Typically, exposure is through a liquid or vapor, with dermal exposure the most dangerous. The lethal dose of inhaled VX is 2.5–3 times higher compared to dermal exposure (Rosman et al., 2014).

Acute manifestations of systemic exposure are dose-dependent and involve nearly every organ system. Defecation, micturition, salivation, diaphoresis, and paralysis can occur (Holstege et al., 1997). In the respiratory tract, increased secretions and bronchoconstriction lead to wheezing and dyspnea, eventually resulting in respiratory failure and death. Initial tachycardia followed by bradycardia and dysrhythmias can occur (Moshiri et al., 2012). Prolonged or severe exposures can result in nervous system manifestations, including confusion, altered mental status, central apnea, and seizures resulting in status epilepticus (Figueiredo, 2018). Those who survive the initial toxidrome can have insomnia, depression, anxiety, and impaired memory.

### 4.1 Ocular complications

The ocular effects of the nerve agent toxidromes are the most sensitive manifestations. Miosis and lacrimation manifest nearly immediately following exposure (McNutt et al., 2020). Interestingly, miosis occurs only with ocular absorption but not with percutaneous exposure (Lukey et al., 2007). Miosis occurs at much lower concentrations than the lethal dose; for example, 3 mg min/m^3^ of sarin (lethal dose 100 mg min/m^3^) and 0.04 mg min/m^3^ of VX (lethal dose 50 mg min/m^3^) cause miosis (Lukey et al., 2007).

Excessive muscarinic stimulation results in ciliary muscle contraction and spasm, leading to blurred vision and myopia with associated ocular pain, headaches, and nausea (Gore, 2020). Eventually, muscarinic desensitization results in a blunted pupillary light reflex. The ocular effects typically resolve completely within weeks (Gore, 2020). There is debate whether these lingering effects are due to a lack of acetylcholinesterase activity or inflammatory irritation of the iris.

### 4.2 Therapeutic considerations

Besides decontamination protocols, nerve agent toxicity is based on pesticide poisoning treatment regimens. Systemic atropine followed by pralidoxime is the therapy of choice (Chai et al., 2017). There is debate as to the appropriate dosage of atropine for prevention of mydriasis and lack of accommodation. There are also drugs that have been tested in animals with better CNS penetration than pralidoxime, however, they are not currently recommended in the management of acute toxicity (Chambers, 2016).

## 5 Knockdown gases–hydrogen cyanide

Hydrogen cyanide has been used as a chemical warfare agent, most notably during World War I and the Iran-Iraq War (Sauer and Keim, 2001; Mégarbane et al., 2003). Given its volatility and rapidly effective reversal agents, large quantities of the gas are needed to be an effective. Although not always present, exposure to cyanide vapor is classically associated with a bitter almond odor and a “cherry-red” skin discoloration (Parker-Cote et al., 2018). Cyanide affects aerobic cellular respiration; thus, symptoms are seen in systems with high metabolic rates. Early neurologic side effects include headache, confusion, and dizziness with seizures and coma in more severe exposures (Alqahtani et al., 2020). As a result of poor tissue oxygenation, acute tachycardia and tachypnea also occur. Later findings include arrhythmias, bradycardia, hypotension, and apnea (Fortin et al., 2010).

### 5.1 Ocular complications

Acute ocular complications of hydrogen cyanide exposure are scarcely reported in the literature due to few survivors receiving an ophthalmologic exam. Acute exposure has been associated with mydriasis and delayed, chronic, severely decreased visual acuity with bilateral optic disc atrophy on exam secondary to optic neuritis (Pentore et al., 1996). A case of bilateral vision loss with a normal fundoscopic exam has been reported as well (Chen et al., 2011). In this case, the physical exam was normal 5 months pre-exposure, but the patient developed visual changes shortly after intoxication. There was no apparent visual pathway lesion on magnetic resonance imaging (MRI) and normal physical examination, optical coherence tomography, retinal nerve fiber layer testing, and color vision testing. The only abnormal finding was visual evoked potentials, which indicated a likely posterior visual pathway lesion (Houston and Hendrickson, 2005).

### 5.2 Therapeutic considerations

Please see the “Therapeutic Approach to Chemical Exposures” section for detailed instructions on decontamination protocols. Rescue breaths are contraindicated in these patients due to the risk of exposure to the provider (Bryson, 1996). Several antidotes exist for acute cyanide toxicity, but hydroxocobalamin is preferred over sodium nitrite and sodium thiosulfate combination management (Hall et al., 2007).

## 6 Caustics–hydrofluoric acid

Hydrofluoric acid (HFA) is a highly corrosive chemical commonly encountered in occupational settings, such as glass etching and industrial and pharmacologic applications (Bajraktarova-Valjakova et al., 2018). It has not been frequently used in warfare or terroristic acts. However, it could potentially be a dangerous chemical weapon, given its unique ability to penetrate deeper into tissue and cause more extensive damage than other acids (McKee et al., 2014). Exposure to HFA can be through vapor inhalation, vapor contact, liquid burns, or ingestion.

With dermal exposure to a >50% concentrated solution, symptoms include intense, immediate pain; pain may not appear until up to 8 h after exposure with less concentrated solutions (Zhang et al., 2016). Intense pain out of proportion to the exam is the hallmark of dermal exposure (McKee et al., 2014). Ulceration and necrosis follow with potential involvement of the underlying tendons and bones. Full-thickness skin necrosis has been noted 1 hour following exposure (Dennerlein et al., 2016).

When exposure occurs via the inhalational route, immediate respiratory tract pain, inflammation, and bleeding occur, and ulceration or septal perforation if severe (Bajraktarova-Valjakova et al., 2018). Laryngitis, laryngotracheitis, and tracheobronchitis can occur and lead to cough, dyspnea, stridor, and wheezing. Pulmonary edema and hemorrhage occur in severe cases. An eventual perforation of the lower airway can lead to pneumothorax.

With ingestion, burns to the oropharynx, esophagus, and gastric mucosa occur rapidly (Balali-Mood and Hefazi, 2005). Nausea, vomiting, and severe pain are common symptoms. Melena, hematemesis, and potential perforation may occur (Bajraktarova-Valjakova et al., 2018). Systemically, fluoride ions in the bloodstream have a direct cardiotoxic effect. However, they also bind magnesium and calcium ions and raise potassium levels leading to a risk of potentially fatal arrhythmias (Vohra et al., 2008).

### 6.1 Ocular complications

Ocular contact, either through liquid or vapor, causes immediate pain, however, pain may be delayed with a low concentration exposure (Hatai et al., 1986). Conjunctivitis with edema and congestion follows pain with subsequent erosion, sloughing, and ulceration of the corneal epithelium (Hatai et al., 1986). Corneal opacification may follow and lead to long-term visual complications, including permanent visual acuity impairment, photophobia, globe perforation, glaucoma, uveitis, and keratitis sicca (Atley and Ridyard, 2015). Delay in treatment leads to worse long-term prognosis (MacKinnon, 1988).

### 6.2 Therapeutic considerations

Please see the “Therapeutic Approach to Chemical Exposures” section for detailed instructions on decontamination protocols and ocular rinsing. With dermal exposure, following copious irrigation, calcium is a first-line chelating agent as it can form inorganic salts with fluoride ions to prevent deep tissue penetration. Following water irrigation, 1% calcium gluconate irrigation may be done for 15–20 min using a Morgan Lens, but variable efficacy has been reported (Rubinfeld et al., 1992; Bentur et al., 1993; Mathieu et al., 2007). Severe necrosis may lead to exposure of the ocular surface, which will require lubrication of the ocular surface with frequent eye drops and ointments. Scarring of the eyelids may require later surgeries such as ectropion repair or more complex reconstructive surgeries such as skin grafts or flaps. Hexafluorine is another safe and effective chelating therapy that binds both free hydrogen and fluoride ions. A case series has shown no sequelae in patients treated with hexafluorine for HFA burns (Soderberg et al., 2004).

## 7 Therapeutic approach for chemical exposures

An important immediate consideration for every agent is decontamination with removal of affected clothing, cleaning the contaminated skin with neutral soap and water, and ocular rinsing for eye exposures. Contaminated clothing should be removed with shears to avoid inadvertent exposure caused by pulling clothing over the face (Balali-Mood and Hefazi, 2005). Ocular rinsing should be done with tap water, normal saline, or lactated Ringer’s solution. It is preferred to use a Morgan Lens or eye irrigator and move the globe in every direction during irrigation. In exposures with alteration in ocular surface pH, irrigation should continue until the ocular surface pH has normalized to a range of 7.0–7.2.

Topical ocular steroids may be used to reduce chemosis and corneal epithelial edema, however, local steroids must be avoided if there is corneal epithelial defects, which may predispose patients to infectious keratitis (Rafati-Rahimzadeh et al., 2019). Administration of topical matrix metalloproteinase inhibitor (MMI) doxycycline has anti-inflammatory properties that can reduce acute and delayed ocular injuries (Kadar et al., 2009). Human amniotic membrane has anti-fibrotic, anti-angiogenic, and anti-inflammatory properties and can be useful for decreasing persistent inflammation and neovascularization (Alió et al., 2005). There has been success with limbal stem cell transplants and corneal keratoplasty for mustard gas keratitis (Javadi et al., 2007; Javadi et al., 2011). In patients with decreased visual acuity due to corneal opacification, penetrating keratoplasty (PKP), lamellar keratoplasty (LKP), or deep anterior lamellar keratoplasty (DALK) are commonly used (Baradaran-Rafii et al., 2011). In addition, limbal stem cell transplantation may be used in patients with persistent epithelial defects, focal corneal thinning and ulceration that do not respond to conservative treatments. Oculoplastics management of cicatricial conditions leading to eyelid malposition should be considered.

Moreover, healthcare provider contamination prevention is paramount while caring for patients. Specifically, proper personal protective equipment should be worn with appropriate training in donning and doffing protocols. These include fluid-impervious gowns, aprons, protective footwear, gloves, chemical-resistant glasses, face shields, and respirators to provide physical barriers to the hands, skin, clothing, eyes, nose, and mouth.

## 8 Conclusion

In this review, we provide a synthesis of the literature on ocular complications and the management of selected chemical agents. However, further investigation is needed to better understand these agents’ acute and chronic complications, as well as appropriate local ophthalmologic and systemic management. Chemical warfare agents continue to remain a threat for military personnel and civilians, especially in areas with political and civil unrest. The ocular effects of these chemical agents are not nearly as well-known as many of their systemic effects. However, the early onset of ophthalmic symptoms requires an assessment of ocular structures in the event of any chemical exposure. Early recognition of the toxidromes is imperative to prevent acute and long-term disabling complications and ocular consequences. A better understanding of these agents will improve our ability to identify and treat both civilians and military personnel in the event of a chemical incident.
